# Effect of Chinese patent medicine Si-Mo-Tang oral liquid for functional dyspepsia: A systematic review and meta-analysis of randomized controlled trials

**DOI:** 10.1371/journal.pone.0171878

**Published:** 2017-02-15

**Authors:** Yunxia Hu, Yu Bai, Zhiyun Hua, Jie Yang, Huahui Yang, Wenjun Chen, Junwei Xu, Zhiqiang Zhao

**Affiliations:** 1 The First College of Clinical Medicine, Nanjing University of Chinese Medicine, Nanjing, Jiangsu, China; 2 Department of Respiration, Zhenjiang Hospital of Traditional Chinese Medicine, Zhenjiang, Jiangsu, China; 3 Department of Intensive Care Unit, Liyang Hospital of Traditional Chinese Medicine, Changzhou, Jiangsu, China; 4 Jockey School of Public Health, Chinese University of Hong Kong, Hong Kong, China; 5 Department of Obstetrics and Gynecology, Zhejiang Hospital of Traditional Chinese Medicine, Hangzhou, Zhejiang, China; Penn State University School of Medicine, UNITED STATES

## Abstract

**Background:**

Si-Mo-Tang oral liquid (SMT) has been widely used to treat functional dyspepsia (FD), but the effectiveness is still controversial. A systematic review and meta-analysis of randomized controlled trials (RCTs) were performed to assess the efficacy and adverse effects of SMT for FD.

**Methods:**

Investigators searched for articles with publication dates to June 21, 2016, from 9 English and Chinese electronic databases. Comparisons were SMT alone or SMT in combination with western medicine as experimental intervention, and western medicine or placebo as the control. We used the Cochrane collaboration tool for assessing risk of bias to evaluate methodologies. Data were synthesized with RevMan 5.3 software. (PROSPERO Registration #CRD42016042003)

**Results:**

Twenty-seven RCTs were included in the review, involving 2,713 participants: 1,383 subjects were in the experimental group and 1,330 in the control group. SMT showed a significant improvement in clinical efficacy (RR 1.14; 95% CI 1.09, 1.20; P<0.00001), but the heterogeneity was also significant (P = 0.0002, I^2^ = 56%). Because of the different interventions in the 2 groups, we performed subgroup and sensitivity analyses to investigate potential sources of heterogeneity. The heterogeneity was smaller after subgroup analysis and the exclusion of a study by Zhu from 2009. The corresponding pooled RR has no obvious change (RR 1.17; 95% CI 1.13, 1.21; P<0.00001). Subgroup analysis by age and drugs administered in control interventions between SMT and western medicine also showed improvement in the efficacy rate. But a data synthesis that excluded high risk of bias in the blinding of participants and personnel showed no significant difference (RR 1.14; 95% CI 0.97, 1.35; P = 0.12). Three studies measured gastric emptying. Two of these studies reported no significant difference between the experimental and control groups, while 1 study showed that SMT reduced the time of gastric emptying. The relapse rate and adverse effects had no difference between 2 groups.

**Conclusions:**

This meta-analysis suggests that SMT is an effective and safe therapy option for patients with FD. However, because of the high clinical heterogeneity, poor quality, high risk of bias and small sample size of some included studies, further standardized large-scale and strictly designed studies are needed.

## Introduction

Functional dyspepsia (FD), a relapsing and remitting disorder, is defined as the presence of 1 or more of the following: pain or burning in the epigastrium, postprandial fullness, early satiation and no evidence of structural disease to explain these symptoms [1]. The global prevalence of FD in the community is between 5% and 11%[1]. FD influences patients’ quality of life, work and other daily activities [2]. It also has substantial financial implications for patients, health care organizations, and society [1,3].

Although great progress has been achieved in the understanding and treatment of FD [4], conventional treatment remains suboptimal. A survey aimed at investigating the current treatments used in functional gastrointestinal disorders (FGIDs) found that treatment of FGIDs was based on symptoms relieved by conventional drugs. However, increasingly, complementary and alternative medicine is used [5].

Traditional Chinese medicine (TCM) utilizes a typically symptoms-based approach, with history-proven therapeutic efficacy [6]. Si-Mo-Tang oral liquid (SMT), a Chinese patent medicine product, is produced by Hansen Pharmaceutical Company and Hunan Wuma Pharmaceutical Company. SMT originated from an ancient and classic formula designed approximately 800 years ago, and is primarily comprised of 4 different kinds of herbs [7]. SMT has been prescribed by TCM practitioners for a longer period of time, and has a good clinical effect. But most studies about the efficacy of SMT has been reported in Chinese, and not readable by any non-Chinese. In addition, the current state of evidence of SMT in treating FD is insufficient. Therefore, we conducted a systematic review and meta-analysis of RCTs to determine whether or not SMT is beneficial to patients with FD.

Because of the widespread application of SMT, participants were included in the review regardless of personal characteristics, like age or sex. Si-Mo-Tang oral liquid alone or in combination with western medicine was used in experimental groups, and a placebo or positive medicine was administered in control groups. Outcomes contained clinical efficacy rates, effects on gastric emptying, relapse rates, safety profiles and adverse events.

## Methods

Reports were in accordance with the Preferred Reporting Items for Systematic Reviews and Meta-Analyses (PRISMA) Statement (S1 Checklist).

### Registration number

We have registered a protocol for this systematic review and meta-analysis in PROSPERO(available from http://www.crd.york.ac.uk/PROSPERO/display_record.asp?ID=CRD42016042003, S1 Protocol).

### Search strategy

For the systematic review and meta-analysis, the following databases were searched: PUBMED(1990 to June 21,2016), EMBASE(1980 to June 21,2016), Cochrane Library, BMJ Clinical Evidence and International Clinical Trials Registry Platform (to June 21,2016), China National Knowledge Infrastructure database (1979 to June 21,2016), Chinese Biomedical Literature database (1978 to June 21,2016), Wanfang database (1990 to June 21,2016), and VIP database (1989 to June 21,2016). The free text search elements were “functional dyspepsia,” “epigastric pain syndrome,” “postprandial discomfort syndrome,” “Pi-Man in Chinese,” “Ji-Zhi in Chinese” and “Wei-Tong in Chinese.” Additionally, the subject heading “dyspepsia” was searched. These terms were combined with the operator AND with the free text term “Si-Mo-Tang.” For example, in the Wanfang database, the combined search terms were “functional dyspepsia or epigastric pain syndrome or postprandial discomfort syndrome or Pi-Man or Ji-Zhi or Wei-Tong” and “Si-Mo-Tang.” Papers published in English and Chinese were evaluated. Only studies with full text were reviewed. Investigators contacted the authors of any articles without full text. A recursive manual search of cited references was performed to identify other relevant studies.

### Data selection

Studies were considered to be eligible for inclusion if they met the following criteria: (i) patients were diagnosed with FD by the ROME I, II, III or IV criteria and on clinical grounds regardless of age, sex, inpatient or outpatient; (ii)the study was performed as a RCT; (iii) Si-Mo-Tang oral liquid alone or in combination with western medicine was compared with placebo or positive controlled studies; (iv) criteria for successful treatment were clearly clarified; (v) treatment lasted for 7 days or more. Studies meeting the following criteria were excluded: duplication (the same data of patients with the same authors published in different journals); lack of information of diagnostic criteria, interventions or outcomes were not defined or suitable; lack of important information of participants’ characteristics; full text could not be obtained; academic fraud or errors; and studies not meeting the inclusion criteria for other reasons.

### Data abstraction

Detailed information abstracted from the studies included the name of first author, year of publication, age of subjects, number of participants, sex, details of intervention, outcome measures, effectiveness and ineffectiveness number (based on the alleviation of symptoms, effectiveness means that the symptoms were relieved more than 30%, ineffectiveness means less than 30%) and duration of treatment. Data were extracted as intention-to-treat analyses (ITT analyses) [8], in which drop-outs were assumed as treatment failures in experimental groups and as efficiency in control groups. Assessment of methodological quality was conducted with the Cochrane Collaboration tool [9].

Eligibility assessment of the literature search, study selection, data abstraction and analysis of study quality were performed independently by YH and YB to avoid bias. Articles were screened repeatedly to confirm, and data were checked for internal consistency. Disagreements between evaluators were resolved by discussing with JY and ZZ.

### Data synthesis and analysis

Meta-analysis was carried out using Review Manager software (version 5.3), provided by the Cochrane Collaboration. Dichotomous data were presented as risk ratio (RR) and continuous outcomes as mean difference (MD), both with a 95% confidence interval (CI). The chi-squared test for heterogeneity was performed before the results were pooled, and heterogeneity was presented as significant when *I*^*2*^was over 50% or *P*<0.1 [10]. A random effect model was used for the meta-analysis if there was significant heterogeneity, and a fixed effect model was used when the heterogeneity was not significant [10]. A subgroup analysis was conducted when the heterogeneity was high, and a sensitivity analysis was done to investigate potential sources of heterogeneity by omitting each trial in turn [11]. Forest plots were used for comparisons, and funnel plots were used to evaluate publication bias.

## Results

### Description of included studies

**Fig 1:**
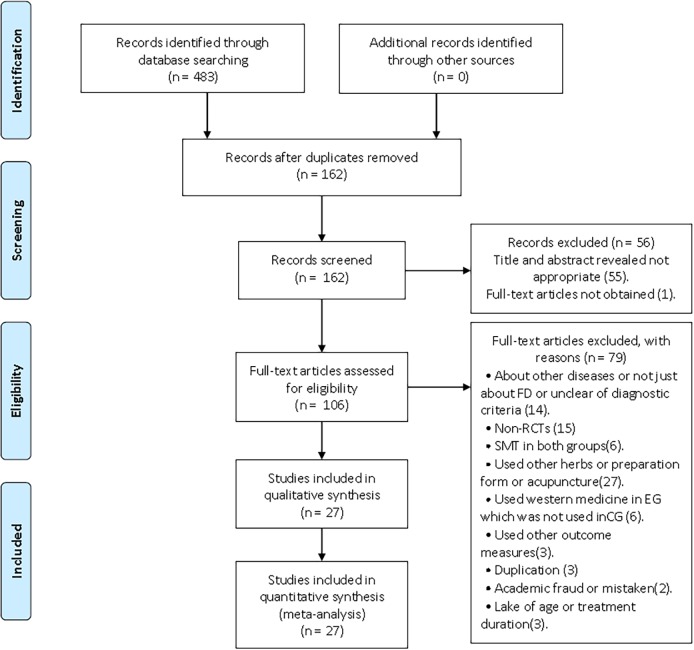
Flow chart of study selection process. PRISMA flow chart of the selection process of SMT-treated FD. SMT = Si-Mo-Tang oral liquid, FD = functional dyspepsia.

A total of 483 studies were identified by both electronic and manual searches of cited references. Of these, 162 articles were duplicated, 55 articles were excluded for inappropriate titles and abstracts, and 1 full-text article could not be obtained. After further screening, a total of 27 studies satisfied the criteria. A flow chart of the study selection process was showed in Fig 1.

In the 27 trials, 1 study was a postgraduate candidate thesis, and the remaining were journal articles. All studies were conducted in China. One was a multi-center study, and the rest were single-center. Total numbers of subjects of the individual studies varied from 30 to 201, with a total of 2,713 participants included. A description of the characteristics of the included studies can be found in Table 1. The interventions were given orally. Si-Mo-Tang oral liquid was given in doses from 3–20 ml three times a day according to age. Other medicines depended on age and weight according to the drug use instructions.

**Table 1:** Summary of the characteristics of the included studies.

	Number	Sex		Age	Intervention		Effectiveness	Ineffectiveness	Duration
First Author	EG/CG	M(EG/CG)	F(EG/CG)	(year)	EG	CG	EG/CG	EG/CG	(day)
Cai 2010[12]	101/100	38/39	63/61	18–65	SMT	Domperidone	81/74	20/26	14
Ji2010[13]	100/100	57/55	43/45	3–12	SMT	Domperidone	96/81	4/19	7
Shen 2012[14]	46/46	/	/	20–63	SMT	Trimebutine+Famotidine	45/37	1/9	28
Tu 2009[15]	55/55	25/28	30/27	4–12	SMT	Domperidone	50/42	5/13	7
Wang 2003[16]	62/62	37/35	25/27	18–72	SMT	Domperidone	58/56	4/6	14
Wang 2000[17]	37/36	17/14	20/22	18–66	SMT	Cisapride	27/28	10/8	28
Wei 1999[18]	69/67	/	/	>18	SMT	Cisapride	57/58	12/9	14
Xiao 2012[19]	30/30	/	/	18–65	SMT	Domperidone	28/26	2/4	14
Zheng 2005[20]	50/48	28/30	22/18	16–68	SMT	Domperidone+Oryzanol	46/35	3/14	14
Zhu 2009[21]	50/50	13/12	37/38	20–68	SMT	Domperidone+Amitriptyline	35/40	15/10	28
Ye 2005[22]	50/50	16/18	34/32	20–58	SMT	Placebo	44/18	6/32	7
Zhou 2014[23]	30/30	/	/	18–65	SMT	Domperidone	27/26	1/3	14
Zhang 2011[24]	33/39	17/18	16/21	17–72	SMT	Cisapride	32/37	1/2	14
Li 2012[25]	42/42	16/19	23/22	18–65	SMT	Domperidone	36/35	3/6	14
Meng 2014[26]	60/60	26/28	34/32	4–13	SMT	Domperidone	55/47	5/13	7
Xie 2016[27]	45/45	24/26	21/19	1–5	SMT+Lactobacillin	Lactobacillin	42/34	3/11	14
Zhang 2016[28]	46/46	21/24	25/22	39–51	SMT+LCBLEC	LCBLEC	40/31	2/9	56
Tian 2014[29]	15/15	9/7	6/8	5–14	SMT+Domperidone suspension	Domperidone	15/13	0/2	14
Wen 2011[30]	108/54	56/28	52/26	5–14	SMT+Domperidone	Domperidone	104/46	4/8	14
Li 2011[31]	96/96	110	82	23–71	SMT+Domperidone	Domperidone	90/72	6/24	28
Zhang 2015[32]	58/55	30/29	28/26	39–48	SMT+Trimebutine	Trimebutine	49/34	9/21	28
Jiang 2010[33]	43/43	15/28	14/29	34–68	SMT+CBSEFGM	CBSEFGM	38/26	5/17	28
Yu 2011[34]	34/34	14/16	20/19	29–41	SMT+Domperidone	Domperidone	31/25	3/9	28
Wu 2014[35]	29/29	16/15	13/14	3–14	SMT+Domperidone	Domperidone	28/24	1/5	14
Hu 2015[36]	30/30	18/14	12/16	23–67	SMT+Mosapride	Mosapride	28/22	2/8	28
Li Lin 2012[37]	35/35	20/21	15/14	46–54	SMT+Domperidone+Amitriptyline	Domperidone+Amitriptyline	33/25	2/10	28
Tang 2010[38]	27/33	14/15	13/18	2-28day	SMT	Lactasin Tablets	24/23	3/19	7

”/” = not mentioned, CG = control group, EG = experimental group, SMT = Si-Mo-Tang oral liquid, LCBLEC: Live Combined Bifidobacterium, Lactobacillus and Enterococcus Capsules, CBSEFGM: Combined Bacillus Subtilis and Enterococcus Faecium Granules with Multivitamins.

### Methodological quality of included studies

**Fig 2:**
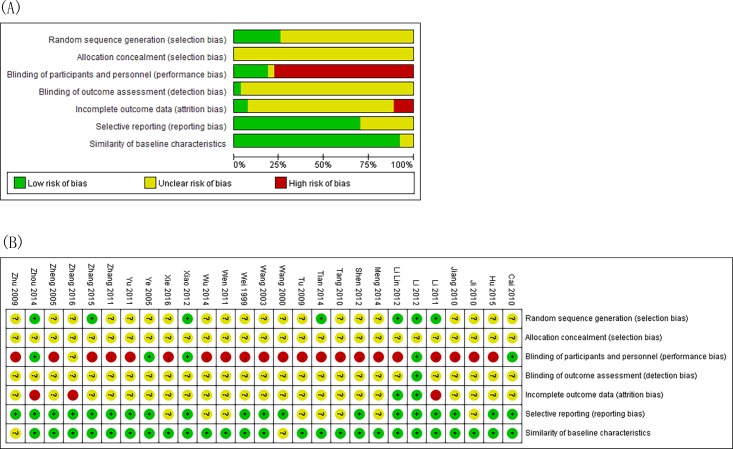
The risk of bias assessment with the Cochrane Collaboration tool. (A)Risk of bias graph. (B)Risk of bias summary.

The risk of bias assessment in the studies is shown in Fig 2. Risk of bias was found across studies for random sequence generation, allocation concealment, blinding of participants, personnel and outcome assessment, incomplete outcome data, selective outcome reporting, and similarity of baseline characteristics [9, 10]. All studies mentioned randomization, but just 6 articles had a detailed description of random sequence generation. None of them discussed allocation concealment. Five studies described blinding of patients, but the other studies did not describe the details. Less study mentioned the details about blinding of outcome assessment. Six studies mentioned follow-up; and 3 of these reported the drop-out or withdrawal information, but did not use ITT analysis. The author reported that characteristics of subjects in different groups have similar baseline (age, sex, race, and disease course) among the studies.

### Primary outcome: Clinical efficacy rate

**Fig 3:**
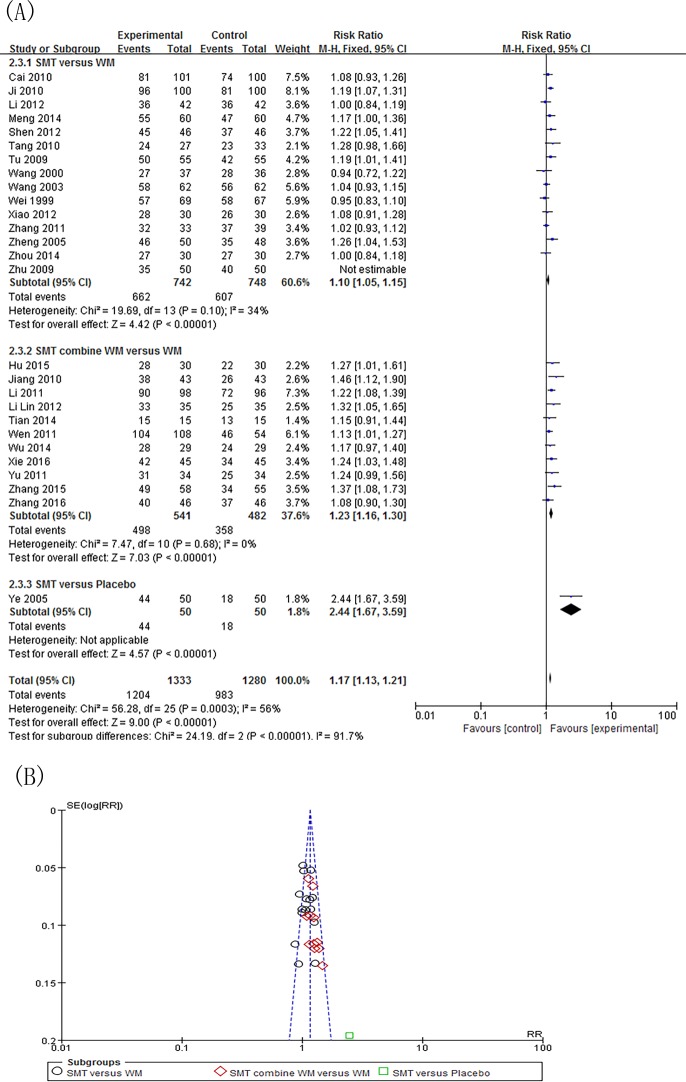
Efficacy rate and publication bias of SMT-treated FD after subgroup of SMT alone or combine with WM versus placebo or WM and sensitivity analysis. (A)Forest plot of comparison: the efficacy rate of symptoms. (B) Funnel plot of publication bias of the included studies. SMT = Si-Mo-Tang oral liquid, WM = western medicine, FD = functional dyspepsia.

**Fig 4:**
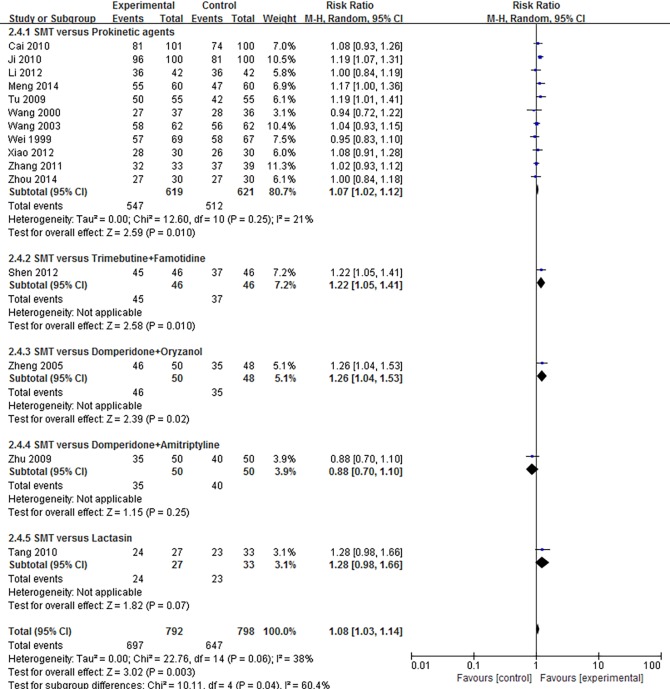
Forest plot of the efficacy rate of SMT-treated FD after subgroup analysis of the different kinds of control interventions among SMT versus WM. SMT = Si-Mo-Tang oral liquid, WM = western medicine, FD = functional dyspepsia.

**Fig 5:**
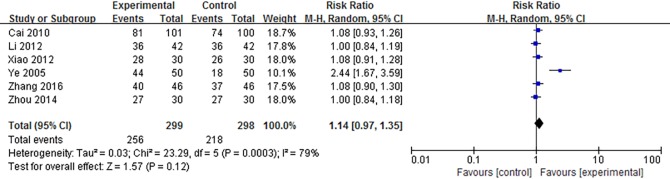
Forest plot of the efficacy rate of SMT-treated FD after excluding poor quality studies. SMT = Si-Mo-Tang oral liquid, WM = western medicine, FD = functional dyspepsia.

Twenty-7 independent studies reported a clinical efficacy rate defined by symptom relief for SMT-treated FD. Participants in the experimental group totaled 1,383, and 1,330 in the control group. SMT showed significant improvement in efficacy (RR 1.14; 95% CI 1.09, 1.20; P<0.00001), but the heterogeneity was significant (*P* = 0.0002, *I*^*2*^ = 56%) (S1 Fig).We performed a subgroup analysis to investigate potential sources of heterogeneity. The included studies fell into 3 subgroups: SMT versus western medicine, SMT versus placebo, and SMT combined with western medicine versus western medicine alone. We then performed a sensitivity analysis, and determined that the Zhu (2009)[21] study was the main origin of heterogeneity in the subgroup analysis. The heterogeneity was smaller after subgroup analysis and exclusion of the study in question. However, the corresponding pooled RR had no obvious change (RR 1.17; 95% CI 1.13, 1.21; P<0.00001) (Fig 3A).The funnel plot demonstrates no apparent asymmetry, suggesting that publication bias is unlikely (Fig 3B). Further examination of the control interventions in the “SMT versus western medicine” subgroups showed that interventions could be categorized by type, such as prokinetic agents, antidepressants, and microbial preparation. Subgroup analysis by different categories of control interventions showed that SMT improved efficacy rate (RR 1.08; 95% CI 1.03, 1.14; P = 0.003) (Fig 4).Subgroup analysis by age also demonstrated the clinical efficacy of SMT (RR 1.16; 95% CI 1.12, 1.20; P<0.00001) (S2 Fig). Due to the importance of blinding of participants and personnel during SMT treatment, we also conducted a data synthesis excluding high risk of bias in the blinding of participants and personnel. Results show that there is no significant difference in the effective rate after SMT treatment (RR 1.14; 95% CI 0.97, 1.35; P = 0.12) (Fig 5).

### Second outcomes

#### Gastric emptying

Three included studies reported gastric emptying, but the data could not be incorporated because Cai (2010)[12] reported efficacy rate, while Ye (2005)[22] referred to the time of gastric emptying, and Zhu (2009)[21] used the emptying rate. Cai and Zhu reported no significant difference between experimental and control group, while Ye reported that SMT significantly reduced the time of gastric emptying.

#### Relapse rate

**Fig 6:**
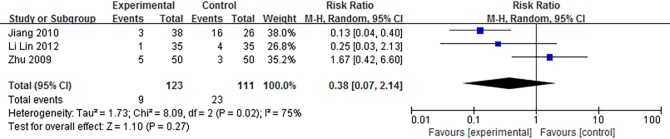
Forest plot of relapse rate of SMT-treated FD between experimental and control group. SMT = Si-Mo-Tang oral liquid, FD = functional dyspepsia.

Three articles discussed a follow-up time. The observation period of Zhu[21] was 8 weeks, Li Lin (2012)[37] was 2 months, and Jiang (2010) [33] was 1 year. The relapse rate was 7.3% for the experimental group and 20.7% for the control group. But as shown in Fig 6, meta-analysis of the 3 studies showed no significant difference between 2 groups.

### Safety profile and adverse events

**Fig 7:**
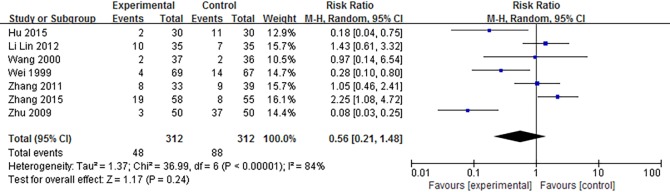
Forest plot of safety profile and adverse events of SMT-treated FD. SMT = Si-Mo-Tang oral liquid, FD = functional dyspepsia.

The safety profile was evaluated in all 27 studies. Eight trials did not mention adverse events. Eleven studies reported no adverse effects in the experimental group during SMT treatment. In the 11 studies of the control group, 7 showed no adverse effects, 3 trials had no mention of adverse events, and 1 research study by Cai[12] revealed that 3 of 41 patients had experienced nausea and dizziness while being treated with Domperidone. A trial by Jiang (2010)[33] reported that 1 of 43 patients appeared to have diluted stool in the experimental group. Another 7 studies showed adverse events in both groups. These adverse events in experimental groups included nausea, dizziness, somnolence, weakness, dry mouth, diarrhea and diluted stool. In the control groups, symptoms also included spastic abdominal pain. The authors, however, said no serious incidents occurred, most of the events did not require special treatment, and were alleviated with time or dose reduction. A meta-analysis of the 7 studies in Fig 7 shows that there was no significant difference between 2 groups (*P* = 0.24). In addition, 23 of the included studies reported that participants in both groups had no organic or systemic disease, such as liver and kidney function damage, while 4 of these studies did not mention the information. Among the 19 studies that reported adverse effects, no case had impact on renal and hepatic function.

## Discussion

This is the first attempt to synthesize clinical data of Si-Mo-Tang oral liquid for functional dyspepsia. In this systematic review, 27 studies were included involving 2713 participants: 1383 versus 1330 between experimental and control group. SMT showed a significant improvement of clinical efficacy rate, and no significant different of relapse rate and adverse effects. Three studies referred gastric emptying, 2 studies reported no significant different between experimental and control group, while 1 showed SMT reduced the time of gastric emptying. The heterogeneity was significant for the primary outcome. We performed a subgroup and sensitivity analysis and found that the Zhu (2009)[21] study may be the main origin of heterogeneity. After subgroup analysis and exclusion of the study, the heterogeneity was effectively decreased while the corresponding pooled RR was not substantially altered. We checked all the included studies carefully and found that there were differences between Zhu and the other groups. In Zhu’s study, besides the experimental and control group in Table 1, there were groups of SMT, Domperidone and Amitriptyline treatment. The third group showed the best efficacy rate. Subgroup analysis by age and different kinds of drugs also showed an improvement in the efficacy rate. But a data synthesis excluding high risk of bias in the blinding of participants and personnel showed no significant difference.

The pathogenesis of FD has not been fully clarified. Numerous mechanisms are involved in the development of FD, including gastric motility and compliance, visceral hypersensitivity, Helicobacter pylori infection, altered gut microbiome and psychosocial dysfunction [39]. It is well known that gastric motility and compliance are a common pathogenesis of FD. Several motility disorders have been reported in patients with dyspepsia. These include delayed gastric emptying, rapid gastric emptying, antral hypomotility, gastric dysrhythmias, and impaired gastric accommodation in response to a meal [39]. Prokinetic agents (such as Domperidone, Cisapride, Mosapride in Table 1) were used widely. The mechanism of SMT is related to its pathogenesis. Ghrelin is a peptide hormone that is involved in gastrointestinal motility and secretion, and therefore, may play a role in functional dyspepsia. Nitric Oxide may play a role as a mediator for ghrelin secretion [40,41]. Kazemi M, Eshraghian A, Hamidpour L, etc. (2015) compared the change of serum ghrelin levels in relation to meal-time between patients with FD and a control group, and found that ghrelin may have an important role in inducing symptoms in FD patients [40]. Several studies explored the possible mechanism of SMT, and found that SMT can regulate the level of Ghrelin and NO in patients with FD [23,42,43].

This Chinese patent medicine is composed of Radix Aucklandiae (Muxiang in Chinese), Fructus Aurantii (Zhiqiao in Chinese), Areca catechu Linn (Binglang in Chinese) and Lindera aggregata (Sims) Kosterm (Wuyao in Chinese) [44].Evidence for the effectiveness of SMT for FD can also be identified in modern pharmacological studies. Guo H, Zhang J, Gao W, etc.(2014) focused on the effects of the methanol extract Radix Aucklandiae (RA ext) on the gastrointestinal tract. They concluded that the mechanism of RA ext might be the root of inhibitory activity. In vitro, RA ext mediated possibly through the combination of Ca^2+^ antagonist and anticholinergic mechanisms, which provides scientific basis for the clinical use of Radix Aucklandiae [45]. Jiang Y, Bai X, Zhu X, etc.(2014) revealed that Fructus Aurantii can enhance gastrointestinal motility by altering 5-HT and vasoactive intestinal peptide expression levels in the rat GI tract [46].Wang Y and Huang T used 95% ethanol for herbal extraction, and found that Areca catechu Linn possessed lower anti-Helicobacter pylori effects [47]. Guo J, Nie Z, Zhang M, etc. (2012) found that the extraction of Lindera aggregata (Sims) Kosterm has the effect of relaxing the isolated ileum of guinea pigs [48]. Although several mechanisms have been proposed, the pathogenesis of functional dyspepsia and the mechanism of SMT-treated FD remains unclear [39].Further studies both in vitro and in vivo need to be conducted to better understand the drug mechanism.

The methodological quality of included studies was showed in Fig 2. Most trails had similar baseline characteristics to ensure the reliability of the research. But there were some flaws in the quality of the included studies. None of the included studies mentioned allocation concealment and fewer provided random sequence generation so the selection bias was high. Few studies mentioned details about the blinding of outcome assessment. Most studies did not describe the details of blinding that may be caused by the different dosage forms between each group, but there were still 5 studies pills and oral liquid. Another flaw was the lack of ITT analysis, which may lead to incomplete outcome data. However, we extracted data as ITT analyses, assuming all drop-outs to be treatment failures, which could reduce the risk of attrition bias [11].

Several possible limitations of this review are worthy of comment. First, while we used a wide range of search strategies to minimize publication bias, some linguistic biases may exist due to language limitations. Second, because of the relatively small sample sizes and the short duration in most studies, the detection of a statistically significant difference between SMT and control group may be limited. It still needs to be demonstrated whether or not the effect size of SMT remains the same when applied in future large-scale studies. Third, the majority of the RCTs had limitations that included the paucity of detailed methodology, non-standardized evaluation of efficacy, and the suboptimal quality of the study design. Therefore, there is a need for further well-designed, long-term, and strictly designed RCTs to explore the effects of SMT for FD.

## Conclusions

This review suggests that SMT is an effective and safe therapy option for patients with FD. However, due to the high clinical heterogeneity, poor quality, high risk of bias and small sample size of some included studies, further standardized preparation, large-scale and strictly designed studies are needed.

## Supporting information

S1 Checklist PRISMA Checklist of SMT for FD. (DOC)

S1 Protocol CRD of SMT for FD. (PDF)

S1 Fig Forest plot of primary outcomes. Efficacy rate of symptoms of SMT-treated FD. SMT = Si-Mo-Tang oral liquid, FD = functional dyspepsia.
(TIF)

S2 Fig Forest plot of the efficacy rate of SMT-treated FD after subgroup analysis by age. SMT = Si-Mo-Tang oral liquid, FD = functional dyspepsia.
(TIF)
