# Supplementary material for: Effect of Chinese patent medicine Si-Mo-Tang oral liquid for functional dyspepsia: A systematic review and meta-analysis of randomized controlled trials
Source: PLoS One. 2017 Feb 15;12(2):e0171878. doi: 10.1371/journal.pone.0171878 (PMC5310891; doi:10.1371/journal.pone.0171878)
Supplement: S1 Protocol — (PDF) [file pone.0171878.s002.pdf]

PROSPERO International prospective register of systematic reviews

Review title and timescale

1 Review title

Give the working title of the review. This must be in English. Ideally it should state succinctly the interventions or exposures being reviewed and the associated health or social problem being addressed in the review.

Effect of SiMoTang oral liquid in functional dyspepsia: a systematic review and meta-analysis of randomized controlled trials

2 Original language title

For reviews in languages other than English, this field should be used to enter the title in the language of the review. This will be displayed together with the English language title.

3 Anticipated or actual start date

Give the date when the systematic review commenced, or is expected to commence.

03/06/2016

4 Anticipated completion date

Give the date by which the review is expected to be completed.

20/08/2016

5 Stage of review at time of this submission

Indicate the stage of progress of the review by ticking the relevant boxes. Reviews that have progressed beyond the point of completing data extraction at the time of initial registration are not eligible for inclusion in PROSPERO. This field should be updated when any amendments are made to a published record.

The review has not yet started ×

Review stage	Started	Completed
Preliminary searches	Yes	Yes
Piloting of the study selection process	Yes	Yes
Formal screening of search results against eligibility criteria	Yes	Yes
Data extraction	Yes	Yes
Risk of bias (quality) assessment	Yes	Yes
Data analysis	Yes	Yes

Provide any other relevant information about the stage of the review here.

Review team details

6 Named contact

The named contact acts as the guarantor for the accuracy of the information presented in the register record.

Dr Yunxia

7 Named contact email

Enter the electronic mail address of the named contact.

huyunxia56@163.com

8 Named contact address

Enter the full postal address for the named contact.

No. 138 Qixia Xianlin Avenue District of Nanjing in Jiangsu,China

9 Named contact phone number

Enter the telephone number for the named contact, including international dialing code.

+8615861128978

10 Organisational affiliation of the review

Full title of the organisational affiliations for this review, and website address if available. This field may be completed as 'None' if the review is not affiliated to any organisation.

None

Website address:

11 Review team members and their organisational affiliations

Give the title, first name and last name of all members of the team working directly on the review. Give the organisational affiliations of each member of the review team.

Title	First name	Last name	Affiliation
Dr	HU	YUNXIA	Nanjing University of Chinese Medicine
Dr	Bai	YU	Nanjing University of Chinese Medicine
Ms	YANG	HUAHUI	The Chinese University of Hong Kong
Professor	ZHAO	ZHIQIANG	Nanjing University of Chinese Medicine
Dr	Yang	Jie	Liyang Hospital of Traditional Chinese Medicine, Changzhou, China
Dr	Hua	ZHIYUN	Zhenjiang Hospital of Traditional Chinese Medicine, Zhenjiang, China

12 Funding sources/sponsors

Give details of the individuals, organizations, groups or other legal entities who take responsibility for initiating, managing, sponsoring and/or financing the review. Any unique identification numbers assigned to the review by the individuals or bodies listed should be included.

National Natural Science Foundation of China NO.81373608

13 Conflicts of interest

List any conditions that could lead to actual or perceived undue influence on judgements concerning the main topic investigated in the review.

Are there any actual or potential conflicts of interest?

None known

14 Collaborators

Give the name, affiliation and role of any individuals or organisations who are working on the review but who are not listed as review team members.

Title	First name	Last name	Organisation details

Review methods

15 Review question(s)

State the question(s) to be addressed / review objectives. Please complete a separate box for each question.

To evaluate the effect of SiMoTang(SMT) on gastrointestinal symptoms, gastric function and gastric sensitivity in humans with functional dyspepsia.

To evaluate the effect of SMT on quality of life in humans with functional dyspepsia.

To evaluate the incidence of adverse events in humans treated with SMT for functional dyspepsia.

16 Searches

Give details of the sources to be searched, and any restrictions (e.g. language or publication period). The full search strategy is not required, but may be supplied as a link or attachment.

An electronic database search will be conducted in PUBMED(1990 to June 21,2016), EMBASE(1980 to June 21,2016),Cochrane Library, BMJ Clinical Evidence and International Clinical Trials Registry Platform (ICTRP)until June 21,2016, CNKI database (1979 to June 21,2016), Chinese Biomedical Literature database (1978 to June 21,2016), Wanfang database(1990 to June 21,2016), and VIP database (1989 to June 21,2016). A free text search will be done to identify articles studying functional dyspepsia using the terms “dyspepsia, functional dyspepsia, epigastric pain syndrome, postprandial discomfort syndrome, Pi-Man, Ji-Zhi or Wei-Tong”. Additionally the subject heading “dyspepsia” will be searched. These terms will be combined with the operator AND with a free text search on “SiMoTang”. Next to an electronic database search, references of included studies will be searched. Only original articles in English and Chinese will be included.

17 URL to search strategy

If you have one, give the link to your search strategy here. Alternatively you can e-mail this to PROSPERO and we will store and link to it.

I give permission for this file to be made publicly available

No

18 Condition or domain being studied

Give a short description of the disease, condition or healthcare domain being studied. This could include health and wellbeing outcomes.

In this review the condition functional dyspepsia will be studied. Functional dyspepsia is an upper abdominal syndrome, which is characterized by the international criteria of the Rome Group. Functional dyspepsia consists of bothersome postprandial fullness, early satiation, epigastric pain or epigastric burning. These symptoms should be present without evidence for a structural disease. In functional dyspepsia changes in gastric function or gastric sensitivity can be present.

19 Participants/population

Give summary criteria for the participants or populations being studied by the review. The preferred format includes details of both inclusion and exclusion criteria.

Studies evaluating humans with functional dyspepsia will be eligible for inclusion. Functional dyspepsia can be defined by the Rome I, II, III or IV criteria and on clinical grounds. The presence of other gastrointestinal disorders, which could cause abdominal discomfort similar to functional dyspepsia should be ruled out.

20 Intervention(s), exposure(s)

Give full and clear descriptions of the nature of the interventions or the exposures to be reviewed

Studies using SMT with any dosage will be considered for inclusion. The duration of the treatment should be at least 7 days.

21 Comparator(s)/control

Where relevant, give details of the alternatives against which the main subject/topic of the review will be compared (e.g. another intervention or a non-exposed control group).

Placebo or positive controlled studies will be included.

22 Types of study to be included

Give details of the study designs to be included in the review. If there are no restrictions on the types of study design eligible for inclusion, this should be stated.

In this review only randomized controlled trials will be included. Crossover studies may be eligible if randomization and blinding took place and if data from separate study groups are available. Only original articles in English and Chinese will be included.

23 Context

Give summary details of the setting and other relevant characteristics which help define the inclusion or exclusion criteria.

24 Primary outcome(s)

Give the most important outcomes.

Change in severity of symptoms measured by a symptom or pain questionnaire.

Give information on timing and effect measures, as appropriate.

Change in gastric function measured by a gastric emptying test or other.

25 Secondary outcomes

List any additional outcomes that will be addressed. If there are no secondary outcomes enter None.

Incidence of adverse events.

Give information on timing and effect measures, as appropriate.

26 Data extraction (selection and coding)

Give the procedure for selecting studies for the review and extracting data, including the number of researchers involved and how discrepancies will be resolved. List the data to be extracted.

27 Risk of bias (quality) assessment
State whether and how risk of bias will be assessed, how the quality of individual studies will be assessed, and whether and how this will influence the planned synthesis.
Two authors will assess the risk of bias of each study individually. For this assessment the Cochrane Collaboration's tool for assessing risk of bias will be used. Any disagreement will be discussed with a third author.

28 Strategy for data synthesis
Give the planned general approach to be used, for example whether the data to be used will be aggregate or at the level of individual participants, and whether a quantitative or narrative (descriptive) synthesis is planned. Where appropriate a brief outline of analytic approach should be given.
Data extracted from the studies will be presented in narrative form and in table form. Additionally when extracted data are suitable a meta-analysis will be done using a fixed-effect/random-effect model. Heterogeneity will be assessed using both chi-squared test and I-squared statistic. Indication of publication bias will be assessed and data about this will be presented using a funnel plot.

29 Analysis of subgroups or subsets
Give any planned exploration of subgroups or subsets within the review. 'None planned' is a valid response if no subgroup analyses are planned.
Dosage of SMT Duration of therapy Differences in gender or age relapse rate

Review general information

30 Type and method of review
Select the type of review and the review method from the drop down list.
Systematic review

31 Language
Select the language(s) in which the review is being written and will be made available, from the drop down list. Use the control key to select more than one language.
Chinese-Simplified

Will a summary/abstract be made available in English?
Yes

32 Country
Select the country in which the review is being carried out from the drop down list. For multi-national collaborations select all the countries involved. Use the control key to select more than one country.
China

33 Other registration details
Give the name of any organisation where the systematic review title or protocol is registered together with any unique identification number assigned. If extracted data will be stored and made available through a repository such as the Systematic Review Data Repository (SRDR), details and a link should be included here.

34 Reference and/or URL for published protocol
Give the citation for the published protocol, if there is one.
Give the link to the published protocol, if there is one. This may be to an external site or to a protocol deposited with CRD in pdf format.

I give permission for this file to be made publicly available
No

35 Dissemination plans
Give brief details of plans for communicating essential messages from the review to the appropriate audiences.
Do you intend to publish the review on completion?
Yes

36 Keywords

Give words or phrases that best describe the review. (One word per box, create a new box for each term)

37 Details of any existing review of the same topic by the same authors
Give details of earlier versions of the systematic review if an update of an existing review is being registered, including full bibliographic reference if possible.

38 Current review status
Review status should be updated when the review is completed and when it is published.
Ongoing

39 Any additional information
Provide any further information the review team consider relevant to the registration of the review.

40 Details of final report/publication(s)
This field should be left empty until details of the completed review are available.
Give the full citation for the final report or publication of the systematic review.
Give the URL where available.
