# Supplementary figures and images for: Effect of Chinese patent medicine Si-Mo-Tang oral liquid for functional dyspepsia: A systematic review and meta-analysis of randomized controlled trials
Source: PLoS One. 2017 Feb 15;12(2):e0171878. doi: 10.1371/journal.pone.0171878 (PMC5310891; doi:10.1371/journal.pone.0171878)

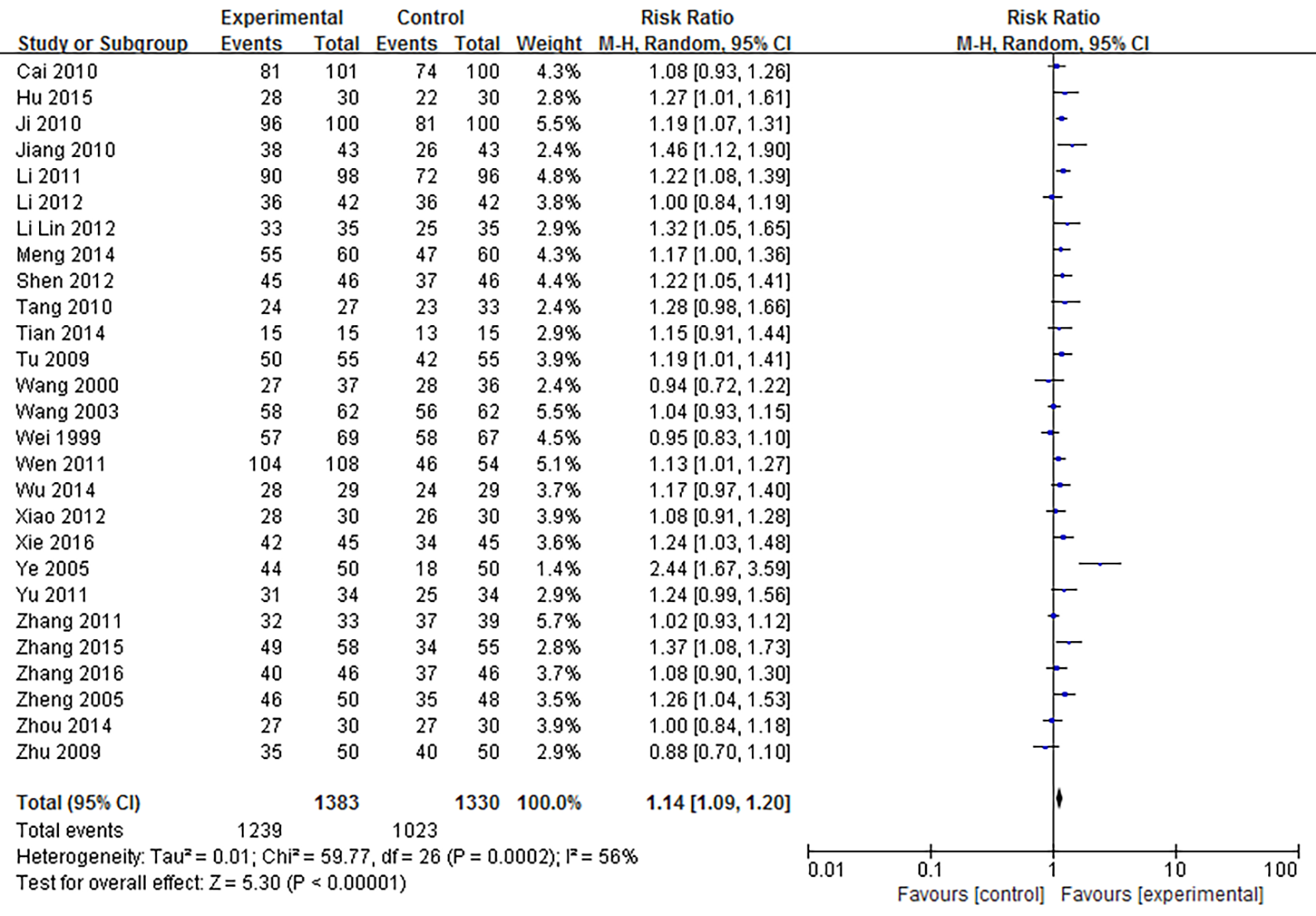

Supplement: S1 Fig — Efficacy rate of symptoms of SMT-treated FD. SMT = Si-Mo-Tang oral liquid, FD = functional dyspepsia. (TIF) [file pone.0171878.s003.tif]

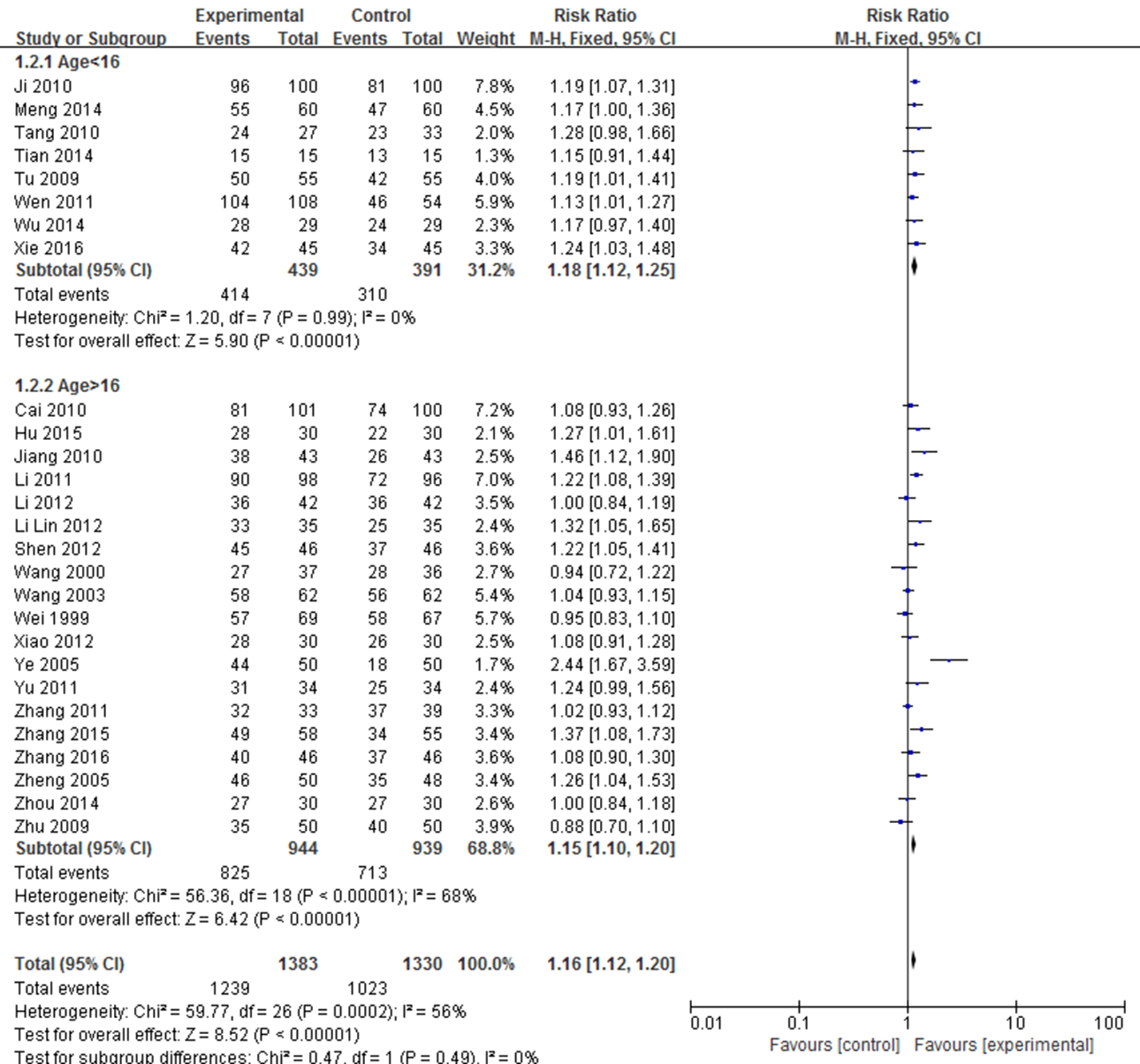

Supplement: S2 Fig — SMT = Si-Mo-Tang oral liquid, FD = functional dyspepsia. (TIF) [file pone.0171878.s004.tif]
